# Parental COVID-19 Vaccine Hesitancy for Children and Its Influencing Factors: A Riyadh-Based Cross-Sectional Study

**DOI:** 10.3390/vaccines11030518

**Published:** 2023-02-23

**Authors:** Mansour Almuqbil, Rama Al-Asmi, Samar AlRamly, Noor Hijazi, Hailah Alotaibi, Ashwaq AlMubarak, Kholoud AlAnezi, Maha Al-Rowaili, Mohammed Al-Yamani, Badr Sami Duwaidi, Dalal Rahil Alshammari, Abdullah Mohammad Alabdulsalam, Jamelah Abdualrhman Almutairi, Fayez Mohammad Alasmari, Syed Mohammed Basheeruddin Asdaq

**Affiliations:** 1 Department of Clinical Pharmacy, College of Pharmacy, King Saud University, Riyadh 11451, Saudi Arabia; 2 Department of Pharmacy Practice, College of Pharmacy, AlMaarefa University, Dariyah, Riyadh 13713, Saudi Arabia; 3 King Abdulaziz Medical City, Riyadh 11426, Saudi Arabia

**Keywords:** COVID-19 vaccine, vaccine hesitancy, children, healthcare, public health

## Abstract

It is well known that vaccination is the best clinical approach for successfully controlling COVID-19 infection. Understanding the disparities in COVID-19 vaccination apprehension among parents in different societies is crucial for effectively implementing COVID-19 vaccination programs. This observational cross-sectional study was carried out in the Riyadh region of Saudi Arabia between February and April 2022. The validated questionnaire was shared with parents who had children between the ages of five and eleven years. The collected data were analyzed using descriptive and inferential statistical methods. Multinomial regression analysis was conducted to determine the factors significantly affecting vaccine-use decisions. Of the 699 participants, 83% of the mothers were between the ages of 35 and 44 years, 67% were university educated, and only 14% were healthcare workers. A large proportion of parents, with an age range of 18–34 years (*p* = 0.001), and those with a higher income group (*p =* 0.014), demonstrated significant vaccine hesitancy. Further, parents who received one or two vaccination doses were significantly (*p* = 0.02) more hesitant than those who received more than two doses of the vaccine. Furthermore, a significantly (*p* = 0.002) high percentage of parents who follow the Ministry of Health (MOH) guidelines for personal preventive measures were hesitant about their children’s vaccination. Concerns about side effects (31.4%) and a lack of safety data (31.2%) on the COVID-19 vaccines were the two most significant reasons for parents to develop vaccine hesitancy. Social media (24.3%), poor perceived immunity (16.3 %), and news articles (15.5%) were the top three contributors to this hesitancy. Vaccinated parents were 8.21 times more likely to be vaccination-hesitant than non-vaccinated parents. Additionally, parents with less education and a COVID-19-positive child at home increased the odds of vaccine hesitancy by 1.66 and 1.48 times, respectively. Overall, one-third of the parents were not prepared to vaccinate their children, and one-quarter of the respondents had not decided about vaccination. This study shows that parents in Riyadh are generally reluctant to vaccinate their children against COVID-19. As social media is a primary source of information for parents, public health professionals should utilize the platform to encourage parents to support vaccine acceptance.

## 1. Introduction

COVID-19, first reported in Wuhan, China, spread rapidly across the world in just one month, causing a global public health emergency [1]. The World Health Organization (WHO) classified the novel coronavirus disease (COVID-19), caused by severe acute respiratory syndrome, coronavirus 2 (SARSCoV2), as a global pandemic on 11 March 2020 [2]. COVID-19, mainly respiratory viruses, can also be transmitted through aerosols made by infected people, including those who are asymptomatic [3]. The clinical features of this disease include asymptomatic or moderate symptoms, such as fever, cough, sore throat, and headache, or severe symptoms, including acute nasal congestion such as pneumonia, respiratory failure requiring mechanical ventilation, multi-organ failure, sepsis, and death [4]. COVID-19 infection caused by the SARS-CoV-2 virus affects only 2% of children and young adults. Children with COVID-19 infection have been reported to have severe COVID-19 infections and deaths, but the numbers are lower than those for adults. As the epidemic progresses, more direct and indirect effects become apparent. The side effects of COVID-19 have had serious repercussions on children’s health and well-being because of truancy, health care, mental health, and social repercussions [5].

Managing COVID-19 requires taking several precautionary measures to protect healthy people from contracting the virus. One of the key methods of managing the transmission of the virus is maintaining a physical distance from other people. This can be conducted by staying at home and only traveling or going out in public when necessary. Additionally, when one is out in public, it is recommended that one should maintain at least two meters distance [6]. Crowded areas should also be avoided as the virus can be transmitted through the air. Another method of avoiding COVID-19 is wearing masks, especially in public. Wearing protective masks prevents aerosols from an infected person from reaching healthy people, especially when they are in close contact [7]. Even though the preventive precautions described above are no longer mandatory, the WHO [8] maintains that vaccination is, without a doubt, the most significant clinical approach for effective disease prevention and control [8]. According to the published research data [9], immunization not only reduces the likelihood of developing an infection but also, on average, resulted in a less severe presentation of COVID-19. As a result, having an effective vaccine available will aid in preventing susceptible people to contracting the virus and providing comprehensive immunity to end the COVID-19 pandemic.

Vaccines are biologics that provide dynamic, adaptable immunity to specific illnesses and contain medications that mimic the germs that cause infection [10]. To stimulate the immune system to make antibodies that recognize and neutralize infectious germs, they are commonly made with killed or attenuated microbes, their surface proteins, or toxins, which are swallowed or inhaled [11]. Vaccines come in various forms, each designed to train our immune systems to combat invading microorganisms. Subunits, recombinant, live-attenuated, inactivated, toxoid, and conjugated vaccines are the four types of vaccinations available [12].

The FDA approved the Pfizer-BioNTech COVID-19 vaccine (mRNA) for emergency use in December 2020, making it the first COVID-19 vaccine. The FDA has since approved the SII/COVISHIELD (*Adenovirus rector*) and AstraZeneca/AZD1222 vaccines (*Adenovirus rector*), the Janssen/Ad26.COV 2.S vaccine (*Adenovirus rector*) developed by Johnson and Johnson, the Moderna COVID-19 vaccine (mRNA), the Sinopharm COVID-19 vaccine (inactivated virus), the Sinovac-CoronaVac vaccine (inactivated virus), the Bharat Biotech BBV152 COVAXIN vaccine (inactivated virus), the Covovax (NVX-CoV2373) vaccine (subunit vaccine), and the Nuvaxovid (NVX-CoV2373) vaccine (subunit vaccine) for emergency use in the prevention of COVID-19 [13].

Children are the primary target demographic for vaccination [14], and many countries worldwide have taken various steps to boost their children’s immunization rates. Despite this, there has been an upsurge in parents refusing or delaying vaccinations for their children. According to a data analysis of the WHO and UNICEF joint report (2015–2017), parental vaccination hesitancy has been observed in more than 90% of nations worldwide [15]. As a result, vaccine hesitancy research has shifted its focus to parents’ attitudes toward immunization [16].

Recent statistics imply that nations such as America may never attain herd immunity. Nevertheless, because children comprise 22% of the American population, engaging children in the vaccination efforts and planning is critical for enhancing community protection against COVID-19 [17,18]. Understanding the disparities in COVID-19 vaccination apprehension across various communities and sociodemographic categories is crucial for identifying those for whom the current COVID-19 vaccine information may be insufficient to increase uptake. Based on this information, vaccine communication and distribution techniques could be customized towards hesitant populations. Several variables can impact a parent’s decision to withhold immunizations from their children. Riyadh is the capital of Saudi Arabia and exhibits a cosmopolitan society with people from different social, religious, and professional backgrounds; it may represent the nation’s mood. Therefore, it was decided to carry out a questionnaire-based cross-sectional study in Riyadh city to explore the prevalence of COVID-19 vaccine hesitancy among parents toward their young children and determine the factors that may influence their decisions.

## 2. Materials and Methods

### 2.1. Study Design, Participants, and Settings

This observational cross-sectional study was carried out in the Riyadh region of Saudi Arabia between February and April 2022. All residents of Riyadh region of Saudi Arabia who were 18 years or older, parents of children between the ages of 5–11 years, and ready to participate were eligible to be included in the study. They were approached at several locations, such as malls, supermarkets, gardens, primary health centers, children’s parks, hospitals, and health camps. They received an online questionnaire link in Google form. At the beginning of the online form, the study’s objectives and informed consent were stated, and parents could choose to participate or decline, making participation voluntary. The participant was requested to register their response by self-administration. The research proposal was approved by the institutional ethical committee of AlMaarefa University with reference number IRB06-06032022-21.

### 2.2. Determination of Sample Size

In 2022, the total population of Riyadh city was estimated to be 7,538,200, as reported by the world population review [https://worldpopulationreview.com/world-cities/riyadh-population (accessed on 20 January 2023)]. Therefore, our study’s sample size was 384, based on the online sample size calculator http://www.raosoft.com/samplesize.html (accessed on 20 January 2023) keeping a 5% margin of error and a 95% confidence level.

### 2.3. Study Questionnaire, Validation, and Pretest

The research team developed the questionnaire with the help of the published literature. Further, it was validated with the help of experts in the field of community health, epidemiology, immunology, pediatric, social health, and pharmacy practice professionals. The questionnaire was translated into Arabic with the help of bilingual professionals by the forward and backward methods. As part of the pilot/pretest, a questionnaire was initially distributed to 30 eligible participants to determine whether a better understanding of any of the study questions was needed. Some questions and statements were rephrased at the end of the pilot study to improve its knowledge. The reliability of the study questionnaire was confirmed by checking the Alpha Cronbach factor, which was found to be 0.82. Finally, a bilingual (Arabic and English) questionnaire was used for the study.

### 2.4. Study Questionnaire

There were four sections in the questionnaire used in the study. All of the sections and items included in each section were required to be completed by the participants. The four sections were sociodemographic characteristics, COVID-19 infection status in the family, COVID-19 vaccination status, and COVID-19 personal preventive measures.

#### 2.4.1. Sociodemographic Characteristics

This section had eleven items to determine the age of the participants, their nationality, gender, marital status, educational level, income range, employment status, whether they work in the healthcare sector, any specific illness of the child, and their child’s routine vaccination status.

#### 2.4.2. COVID-19 Infection Status

This section explored whether the child/children and any family member was/were ever infected with the COVID-19 infection (yes or no) and the severity level (asymptomatic, mild symptoms, moderate symptoms, severe symptoms).

#### 2.4.3. COVID-19 Vaccination Status

This section recorded the COVID-19 vaccination status of the child and the family. It also documented the number of jabs that the parents and their children had received. Further, parents who were not ready for their children’s vaccination were inquired about the possible reasons for avoiding it. The reasons that were put forth to the participants were: (a) Inadequate data about the safety of a new vaccine; (b) I am against vaccines in general (or I avoid medications whenever possible); (c)Vaccine administration is painful or inconvenient; (d) My child already had a COVID infection; (e) A concern about the adverse effects of the vaccine; (f) A concern of the vaccine being ineffective from COVID mutations; (g) Prior adverse reaction to the vaccine; (h) I perceive my child as not at high risk to acquire COVID-10 infection; and (i) I perceive my child as not at high risk to develop complications if he/she contracts COVID-19. Those parents who were reluctant to vaccinate their children were also inquired about the influencing factors for their decision. The following list was presented to them for the selection of relevant factors: (a) Social media; (b) Religious belief; (c) Family members; (d) News articles; (e) My child’s poor perceived immunity; (f) My dislike of the vaccine; and (g) My colleagues.

#### 2.4.4. COVID-19 Personal Preventive Measures

This section was intended to determine the parents’ regular practice towards personal preventive measures during the pandemic. They were inquired about family commitment, the use of protective items, and avoiding crowded areas during infection. The recording was conducted using a Likert scale ranging between every time, often, sometimes, and never. With the inclusion of four extreme possibilities, we picked this scale to eliminate the influence of a neutral choice [https://tinyurl.com/37bkm9nr (accessed on 11 February 2023)].

### 2.5. Data Analysis

The data collected were entered into the SPSS IBM statistical package (version 25). Univariate descriptive analysis of the socio-demographic characteristics of the study sample and bivariate analysis, using the Pearson Chi-square test, were conducted. The factors responsible for influencing the parents’ decision on vaccine hesitancy were determined using stepwise binary regression analysis, followed by multinomial regression analysis, to calculate the odds ratio. A P-value of less than 0.05 was significant.

## 3. Results

### 3.1. Sociodemographic Characteristics of the Participants

The study’s 699 participants included 83% mothers, and the overwhelming majority of them were between the ages of 35 and 44 (42%) and 18 and 34 (37%) (Table 1). A large percentage of surveyors (93%) were married and living with their spouses, and one-third (67%) were well-educated (university educated). More than half of the surveyors said their family income was between 5000 and 10,000 Saudi riyals (1333 to 2666 US$) per month. Saudi nationals made up more than one-third of the surveyors, while only 14% were healthcare employees (physicians, pharmacists, nurses, and others). Moreover, almost half (46%) of the participants were employed, and 45% were solely doing household work.

As given in Table 2, only 6% and 8% of the children to whom the participants referred in this study were suffering from organic/psychological illness and chronic diseases, respectively. Further, 96% of the children received routine vaccinations.

### 3.2. Comparison of Sociodemographic Characteristics and Vaccine Hesitancy Status

Compared to the other age groups, a significantly (*p* = 0.001) larger proportion of younger parents (18–34 years) had reservations about their children receiving the COVID-19 vaccine. Furthermore, compared to parents from other income categories, the parents from higher income groups indicated a significant (*p* = 0.014) reluctance to their children receiving COVID-19 immunization. However, marital status, employment position, nationality, educational level, and whether they were a healthcare worker had no significant impact on their willingness or hesitations to vaccinate their children against COVID-19 (Table 3).

### 3.3. Comparison of COVID-19 Infection Status and Vaccine Hesitancy

As shown in Table 4, a parent’s decision to vaccinate their child is based on their child’s infection status. The parents’ aversion to immunization was significantly low (*p* = 0.010) for children who had never been infected with COVID-19. Moreover, the parents of children with more severe symptoms had especially (*p* = 0.044) lower vaccine resistance. The presence of COVID-19 infection in any family members did not affect the parents’ attitude toward their children being vaccinated.

### 3.4. Comparison of Child Vaccine Hesitancy with Vaccination Status of the Family

Table 5 shows that a significantly (*p* = 0.02) high percentage of parents who had undergone vaccination were hesitant to vaccinate their children against COVID-19. In addition, a considerable (*p* = 0.000) percentage of people who had reservations about their children’s vaccination had only received one or two doses of the vaccine; those who had had three doses had significantly less resistance.

### 3.5. Comparison of Vaccine Hesitancy with Personal Preventive Measures

Table 6 compares the parents’ willingness to vaccinate their children with their use of personal preventive measures. More than half of the parents who took part in this survey and claimed (medium, very, very highly) to take individual preventative measures as recommended by the MOH showed significant (*p* = 0.043) opposition to vaccination for their children. These parents consistently use face masks (*p* = 0.016), keep a safe distance (*p* = 0.001), and avoid going into crowded areas (*p* = 0.002), but they have significant reservations about their children receiving the COVID-19 vaccine.

### 3.6. Comparison of Vaccine Hesitancy with Child Health Status

There was no noticeable impact of the children’s chronic or psychological illness on their parents’ unwillingness to vaccinate them against COVID-19 (Table 7).

### 3.7. Reasons for Parents’ Decision on Children’s Vaccine Hesitancy

**Figure 1:**
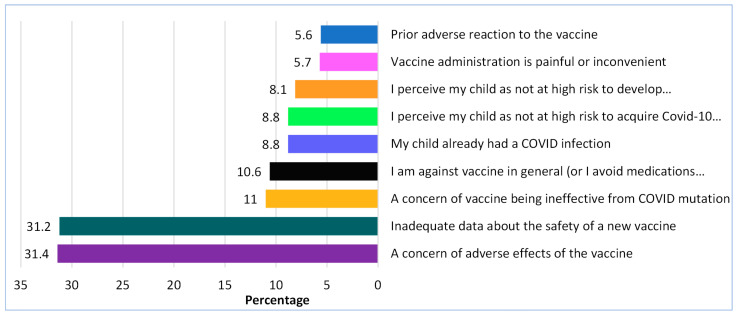
Reasons for parents’ decision on children’s vaccine hesitancy.

Concerns about side effects (31.4%) and a lack of safety data (31.2%) on the COVID-19 vaccinations were the top two reasons for parents in this study developing resistance to immunizing their children (Figure 1). Other reasons included the possibility that the COVID-19 vaccine would be ineffective against virus mutations, personal anti-vaccination sentiment, previous COVID-19 infection, the expectation that their child would not develop complications even if infected, pain at the injection site, and a history of adverse reactions to any vaccine.

### 3.8. Factors Influencing the Parents’ Decision on Vaccine Hesitancy

**Figure 2:**
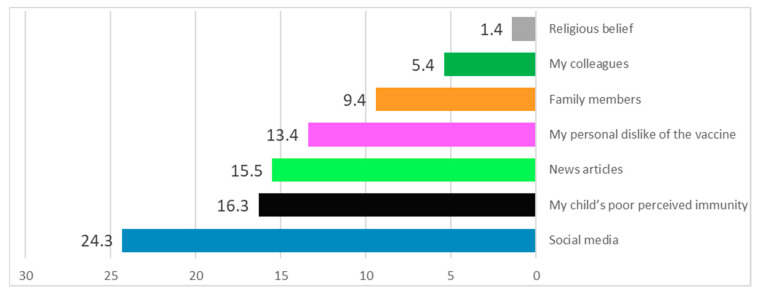
Factors influencing the parents’ decision on vaccine hesitancy for their children.

Social media (24.3%) played a significant role in developing parental apprehension about the COVID-19 vaccine. The second and third factors were poor perceived immunity (16.3%) and news articles (15.5%), which were responsible for parents’ aversion to their children’s vaccinations. Finally, the fourth factor leading to parents’ vaccine concern for their children was personal dislike of vaccines. Furthermore, other family members (9.4%), colleagues (5.4%), and religious beliefs influenced some parents’ decisions (1.4%) (Figure 2).

### 3.9. Stepwise Linear Regression Analysis

The role of the independent factors on the dependent variable was determined using bivariate stepwise regression analysis (Table 8). First, the parent’s willingness to vaccinate their children was kept as the dependent variable. In contrast, the participants’ age, marital status, employment status, nationality, family income, healthcare worker category, psychological status of the child, the status of chronic disease, COVID-19 status of child and family, severity status of COVID-19 if infected by child/family, vaccination status of parents, doses of vaccination, and commitment to MOH regulations on preventive measures were kept as the independent variables. Five independent variables were determined to be significant. They were added one at a time to better understand the impact of the first independent factor in the presence of the others. The most crucial factor was the COVID-19 vaccine doses received by the parents. Compared to parents who received more than two doses of the COVID-19 vaccine, parents who received up to two doses showed 2.542 times more reluctance to vaccinate their children. In the second step, another relevant factor, ‘the age of the participants’, was incorporated, and the value of the COVID-19 vaccination factor was reduced to 2.47 (Odds ratio-OR). The third component was low educational level, introduced in the third step, followed by parent vaccination and child COVID-19 positive in steps 4 and 5, respectively. Participants with a low educational level (less than a bachelor’s degree), those who had been vaccinated, and those whose children had been infected with COVID-19 had considerably higher reservations about their children receiving the COVID-19 vaccine.

### 3.10. Multinomial Regression Analysis

Table 9 shows the results of the study variables’ multinomial regression analysis. The dependent factor, parents’ willingness to vaccinate their children, was strongly influenced by the participants’ age, educational status, COVID-19 vaccine doses, parents’ vaccination status, and the child’s COVID-19 positive status. Overall, the parents who were vaccinated had the highest level of opposition to their children’s vaccination (OR-8.213, *p* = 0.046), followed by those with less than a university education (OR-1.660, *p* = 0.009), those who had children with previous COVID-19 positivity (OR-1.483, *p* = 0.030), those younger than 44 years (OR-1.197, *p* = 0.009), and those who had only one or two doses of the COVID-19 vaccine (OR-1.047, *p* = 0.000).

## 4. Discussion

This study determined the prevalence of vaccination hesitancy among Riyadh city parents regarding their children receiving the COVID-19 vaccine. According to our findings in this study (up to April 2022), more than half of the parents needed more time to be ready or were unsure whether to vaccinate their children against COVID-19. This trend of vaccination apprehension is more common among parents who have only had one or two doses of the vaccine, are less educated, have children who have previously been infected with COVID-19, and are relatively young (less than 44 years). In addition, most parents who oppose the vaccination are apprehensive about the perceived adverse effect and safety of the approved vaccines for children.

Previous studies have found that hesitant parents are reluctant to vaccinate their children even for routine vaccinations and that 25.8% of parents still need to be ready for the annual influenza vaccine [19]. As a result, a similar barrier was envisaged for COVID-19 immunization, allowing policymakers and strategists to address vaccine skeptics’ concerns [20]. As a result, healthcare personnel were included in the CDC’s effort to reinforce or strengthen the streamlining of accurate information to the general population. As is the case in any other society, Saudi Arabia faces challenges in vaccinating children. According to previous research from Saudi Arabia [21], 61.9% (up to November 2021) were hesitant to vaccinate. Our research shows that 33.8% are unwilling to vaccinate their children against COVID-19, while 24.7% are undecided, totaling a worrisome 58.5% vaccine aversion. This indicates that there has been no significant improvement in the vaccine acceptance rates between November 2021 and April 2022. Only 35% and 33% of hesitancy are reported in studies from Qatar [22] and Chicago [23], respectively, although both types of research included parents with children older than 11. According to an article from Israel with a similar sample population, 43% of people were hesitant [24]. However, recent research from other countries has indicated lower percentages of vaccination hesitancy. For example, studies from China, Vietnam, and Italy found that roughly 26%, 21%, and 18% of parents were hesitant to vaccinate their 5–17 year old, 3–17 year old, and 12–18 year old children [25,26,27]. These findings point to a decrease in parental apprehension over immunization. Although there was a trend toward higher hesitancy when parents of children under the age of 12 were included in the research, the results were inconsistent between nations, indicating that the actual rate of reluctance varies.

Previous research has linked conspiracy theories, fake news, and social media to vaccine apprehension [28,29]. Our study discovered that social media contributed more than any other component to the public’s development of parenteral fear regarding COVID-19 immunization by circulating false news or insufficient facts. In more than half of the instances, other factors contributed to vaccine reservations, including poor perceived immunity, news publications, insufficient knowledge, transmission from colleagues and friends, and religious beliefs. Vaccination apprehension among the general population is usually due to a lack of awareness about vaccine safety profiles [30]. The top two reasons for the parents in this study developing reluctance to immunizing their children were concerns about side effects (31.4%) and a lack of safety evidence (31.2%) on the COVID-19 vaccines (Figure 1). Other explanations were personal anti-vaccination attitudes, previous COVID-19 infection, the belief that their child would not develop complications even if infected, pain at the injection site, and a history of adverse vaccine reactions. Beyond a rational “risk vs. benefit” examination, an individual’s vaccination decisions are influenced by various factors. They should thus be viewed as a continuum rather than a binary (anti-vaccine vs. pro-vaccine) viewpoint [31]. The continuous nature of vaccine acceptance allows us to obtain a better picture of vaccine opponents, who are more diverse than one might think.

Our study found several demographic characteristics playing a significant role in developing vaccine hesitancy. The WHO have noted that this as a major threat to global health from preventable illnesses [32]. Compared to other age groups, a significantly (*p* = 0.001) larger proportion of younger parents (18–34 years) had reservations about their children receiving the COVID-19 vaccine. Our study findings are similar to two other studies reported recently. In a Turkish survey, willingness to allow their children to receive the COVID-19 vaccine was higher among parents aged 40 or older compared with those aged 18–29 years old [33], and according to a study conducted in Brazil, younger age participants were associated with a refusal of the COVID-19 vaccine for their children [34]. Young parents may be influenced easily by fake news or misinformation and therefore develop hesitancy about children’s vaccination. On the contrary, a study [35] from China reported no significant impact of the parents’ age on developing children’s vaccine hesitancy. Nevertheless, educating young parents more convincingly about successful vaccinations is important. Further, the low level of education of the parents was a significant factor for their reservations about children’s vaccination. There are variations in the published literature on this aspect. For example, a study [36] shows an inverse correlation between the parent’s educational level and vaccine hesitancy, while another study [37] did not find any correlation between these factors.

This study has a few limitations, but it also offers some advantages. The study’s cross-sectional design and reliance on self-reported data make it impossible to track the participants’ ultimate vaccination decisions for their children. In addition, we could not quantify the study’s response rate as the online questionnaire approach we employed did not allow us to tally the number of invitations sent to parents. As a result, it needs to be clarified whether the group that did not participate in the study had different outcomes than the cohort that did, which could indicate selection bias. Furthermore, the study’s over-representation of females could be a contributing issue. As females were more hesitant to vaccinate their children than males and because females were overrepresented in the survey, our findings may exaggerate the actual hesitation rate. Other studies have evaluated vaccine reluctance among parents of all children under 18 years, while this study focused on parents of 5–11 year old children.

## 5. Conclusions

Although the COVID-19 vaccination has been approved for use in children aged between 5 and 11 years, parental apprehension was still widespread in Saudi Arabia’s Riyadh region. Inaccurate information and fake news have contributed to the development of vaccine-related fear. Parents are particularly concerned about the vaccine’s safety and efficacy. As parents rely on social media for information, health officials should take advantage of the platform and convey accurate information to the public to increase acceptability.

## Figures and Tables

**Table 1:** Sociodemographic characteristics.

Characteristics	Variables	Frequency	Percentage
1. Participants’ status	a. Mother	581	83.1%
	b. Father	101	1.4%
	c. guardian	17	2.4%
2. Age	a. 18–34	256	36.6%
	b. 35–44	292	41.7%
	c. 45–55	137	19.6%
	d. 55–64	11	1.6%
	e. above 64	3	0.4%
3. Marital status	a. Married	648	92.7%
	b. single parent	40	5.7%
	c. widow	11	1.6%
4. Educational level	a. High school or less	232	33.1%
	b. College—university	398	56.9%
	c. High degree	69	9.9%
5. Income (Saudi Riyal)	a. Less than 5000 (Low income)	14	2.0%
	b. 5000–10,000 (Middle income)	389	55.6%
	c. more than 10,000 (High income)	36	5.1%
	d. Prefer not to answer	260	37.2%
6. Nationality	a. Saudi	448	64.0%
	b. Non-Saudi	251	36.0%
7. Employment	a. Unemployed	14	2.0%
	b. Employed	325	46.5%
	c. Housewife	315	45.0%
	d. Retired	12	1.7%
	e. Student	33	4.7%
8. Healthcare worker category	a. Physician	24	3.4%
	b. Pharmacist	20	2.8%
	c. Nurse	27	3.9%
	d. another healthcare worker	30	4.3%
	e. I am not a healthcare worker	598	85.5%

**Table 2:** Health status of the child.

Characteristics	Frequency	Percentage
1-Organic or psychological illness		
a. Yes	40	6%
b. No	659	94%
2-Chronic diseases		
a. Yes	54	8%
b. No	645	92%
3-Received routine vaccination		
a. Yes	674	96%
b. No	27	4%

**Table 3:** Comparison of sociodemographic characteristics with willingness for child vaccination.

Characteristics	Willingness for Child Vaccination, n (%)			*p* Value
	Yes	No	Total	
1-Participants’ status				0.125
(a) Mother	232 (40)	349 (60)	581	
(b) Father	51 (50)	50 (50)	101	
(c) Guardian	8 (47)	9 (53)	17	
2-Age (years)				
(a) 18–34	86 (34)	170 (66)	256	0.001
(b) 35–44	125 (43)	167 (57)	292	
(c) 45–55	71 (52)	66 (48)	137	
(d) 55–64	6 (55)	5 (45)	11	
(e) above 64	3 (100)	0 (0)	3	
3-Marital status				
(a) Married	266 (41)	382 (59)	648	0.520
(b) Single parent	20 (50)	20 (50)	40	
(c) Widow	5 (45)	6 (55)	11	
4-Education level				
(a) High school or less	109 (47)	123 (53)	232	0.124
(b) College—University degree	154 (39)	244 (61)	398	
(c) Higher degree	28 (41)	41 (59)	69	
5-Family income				
(a) Less than 5000 SR * (Low income)	6 (43)	8 (57)	14	0.014
(b) 5000–10,000 SR (Middle income)	156 (40)	233 (60)	389	
(c) More than 10,000 SR (High income)	7 (19)	29 (81)	36	
(d) Prefer not to answer	122 (47)	138 (53)	260	
6-Nationality				
(a) Saudi	197 (44)	251 (56)	448	0.093
(b) Non-Saudi	94 (37)	157 (63)	251	
7-Employment				
(a) Unemployed	7 (50)	7 (50)	14	0.446
(b) Employed	145 (45)	180 (55)	325	
(c) Housewife	119 (38)	196 (62)	315	
(d) Retired	5 (42)	7 (58)	12	
(e) Student	15 (45)	18 (55)	33	
8-Healthcare worker category				
(a) A Physician	8 (33)	16 (67)	24	0.879
(b) A Pharmacist	7 (35)	13 (65)	20	
(c) A Nurse	12 (44)	15 (56)	27	
(d) Other health care worker	13 (43)	17 (57)	30	
(e) I am not a healthcare worker	251 (42)	347 (58)	598	

* SR: Saudi Riyal.

**Table 4:** Comparison of willingness for child vaccination with COVID-19 infection status.

Characteristics	Willingness for Child Vaccination, n (%)			*p* Value
	Yes	No	Total	
(1) COVID-19 infection status of the child				
(a) Yes	174 (61)	111 (39)	285	0.010
(b) No	201 (49)	213 (51)	414	
(2) child COVID situation				
(a) Asymptomatic	13 (34)	25 (66)	38	0.044
(b) Mild symptomatic	84 (50)	85 (50)	169	
(c) moderate symptoms	32 (45)	39 (55)	71	
(d) severe symptoms	11 (55)	9 (45)	20	
(3) family COVID-19 infection				
(a) Yes	223 (42)	307 (58)	530	0.673
(b) No	68 (40)	101 (60)	169	
(4) family COVID-19 situation				
(a) Very mild, asymptomatic	15 (56)	12 (44)	27	0.376
(b) Mild	49 (45)	60 (55)	109	
(c) Moderate	103 (43)	138 (57)	241	
(d) Severe	34 (33)	69 (67)	103	
(e) Very severe	16 (40)	24 (60)	40	
(f) Death	7 (50)	7 (50)	14	

**Table 5:** Comparison of willingness for child vaccination with vaccination status of the family.

Characteristics	Willingness for Child Vaccination, n (%)		Total	*p* Value
	Yes	No		
1-Participant’s vaccination status				
(a) Yes	290 (43)	392 (57)	682	0.002
(b) No	1 (6)	16 (94)	17	
2-Participant’s vaccination doses				
(a) One dose	4 (24)	13 (76)	17	0.000
(b) Two doses	76 (31)	173 (69)	249	
(c) Three doses	210 (50)	206 (50)	416	
(d) Not applicable	1 (6)	16 (94)	17	
3- Participant’s partner vaccination status				
(a) Yes	282 (42)	394 (58)	676	0.805
(b) No	9 (39)	14 (61)	23	

**Table 6:** Comparison of willingness for child vaccination with personal preventive measures.

Characteristics	Willingness for Child Vaccination, n (%)			*p* Value
	Yes	No	Total	
1-Family commitment measures				
(a) Rarely committed	7 (21)	26 (79)	33	0.043
(b) Slightly committed	52 (51)	49 (49)	101	
(c) Medium commitment	131 (42)	184 (58)	315	
(d) Highly committed	68 (40)	101 (60)	169	
(e) Very Highly committed	33 (41)	48 (59)	81	
2-Face Mask				
(a) Every time	137 (38)	223 (62)	360	0.016
(b) Often	65 (39)	103 (61)	168	
(c) Sometimes	82 (53)	74 (47)	156	
(d) Never	7 (47)	8 (53)	15	
3-Other Measures				
(a) Every time	121 (36)	215 (64)	336	0.001
(b) Often	96 (46)	112 (54)	208	
(c) Sometimes	70 (52)	65 (48)	135	
(d) Never	4 (20)	16 (80)	20	
4-Avoiding a crowded area				
(a) Every time	100 (42)	139 (58)	239	0.002
(b) Often	39 (27)	104 (73)	143	
(c) Sometimes	102 (53)	90 (47)	192	
(d) Never	50 (40)	75 (60)	125	

**Table 7:** Comparison of willingness for child vaccination with child health status.

Characteristics	Willingness for Child Vaccination, n (%)			*p* Value
	Yes	No	Total	
1-Child/children organic or psychological illness				
(a) Yes	15 (37)	25 (63)	40	0.585
(b) No	276 (42)	383 (58)	659	
2-Child/children chronic disease				
(a) Yes	23 (43)	31 (57)	54	0.881
(b) No	268 (42)	377 (58)	645	

**Table 8:** Stepwise Linear regression analysis.

Variables		*p* Value	Odds Ratio	95% Confidence Interval	
				Lower	Upper
Step 1	Parent COVID-19 Vaccine doses	0.000	2.542	1.843	30506
Step 2	Parent doses	0.000	2.471	1.788	3.415
	Age of participants	0.006	0.594	0.410	0.861
Step 3	Educational status	0.015	0.660	0.472	0.923
	Parent doses	0.000	2.619	1.884	3.641
	Age of participants	0.015	0.628	0.432	0.915
Step 4	Educational status	0.012	0.650	0.464	0.911
	Parent doses	0.000	2.411	1.728	3.365
	Parent vaccine	0.040	0.117	0.015	0.908
	Age of participants	0.009	0.600	0.410	0.879
Step 5	Educational status	0.011	0.646	0.461	0.906
	Parent doses	0.000	2.412	1.726	3.370
	COVID 19 positive Child	0.025	0.695	0.506	0.955
	Parent vaccine	0.045	0.123	0.016	0.951
	Age of participants	0.009	0.602	0.411	0.883

**Table 9:** Multinomial regression analysis.

Vaccine Willingness-R	*p* Value	Odds Ratio	95% Confidence Interval	
			Lower Bound	Upper Bound
Parent vaccine	0.046	8.213	1.038	65.014
Educational status	0.009	1.660	1.137	2.424
COVID 19 positive Child	0.030	1.483	1.038	2.118
Age of participants	0.009	1.197	0.405	1.881
Parent doses	0.000	1.047	0.313	1.638

## Data Availability

The data presented in this study are available on request from the corresponding author. The data are not publicly available to keep confidentiality of the participants.
